# Resveratrol as a Natural Autophagy Regulator for Prevention and Treatment of Alzheimer’s Disease

**DOI:** 10.3390/nu9090927

**Published:** 2017-08-24

**Authors:** Xianjuan Kou, Ning Chen

**Affiliations:** Tianjiu Research and Development Center for Exercise Nutrition and Foods, Hubei Key Laboratory of Sport Training and Monitoring, College of Health Science, Wuhan Sports University, Wuhan 430079, China; kouxianjuan@126.com

**Keywords:** autophagy, resveratrol, Alzheimer’s disease, microRNA, therapeutic target

## Abstract

Alzheimer’s disease (AD) is one of the most common neurodegenerative disorders over the age of 65 years old. Although several underlying mechanisms for explaining the pathogenesis of AD are elucidated, the effective supplements or drugs for the intervention of AD are still limited. Recently, impaired autophagy associated with miRNA dysfunction has been reported to involve in aging and aging-related neurodegenerative diseases. Thus, the activation of autophagy through effectively regulating miRNAs may become a potential target for the prevention or treatment of AD. Mounting evidence from in vitro and in vivo AD models has demonstrated that resveratrol, one of polyphenolic compounds, can exert neuroprotective role in neurodegenerative diseases especially AD. In this review, the regulation of miRNAs and autophagy using resveratrol during the prevention and treatment of AD are systematically discussed, which will be beneficial to establish a target for the direct link between pharmacological intervention and AD in the future.

## 1. Introduction

Alzheimer’s disease (AD) is a progressive neurodegenerative disorder, and its prevalence is strongly correlated with aging. Progressive loss of memory and cognitive functions are the major features of AD. The pathogenesis of AD is complex due to the influence from genetic and environmental factors. Although many AD-related hypotheses have been proposed, the causes and pathogenesis of AD are still unclear at present. Abnormal or mis-folded proteins aggregated in cytoplasmic, nuclear and extracellular inclusions cause organelle damage and synaptic dysfunction in the nervous system [1]. Amyloid-β (Aβ) peptide aggregation resulted from imbalanced Aβ production and disordered Aβ clearance may play a crucial role in the development and progression of AD. Neurons are post-mitotic cells and aggregation-prone proteins in neurons cannot be attenuated by cell division. Thus, neurons require well-regulated protein quality control systems. It is well known that ubiquitin-proteasome system (UPS) mainly degrades short-lived and soluble proteins. However, the autophagy-lysosome system digests long-lived and abnormal protein complexes and organelles. Hence, autophagy deficiency and/or autophagy dysfunction due to reduced lysosomal function in neurons contributes to the pathogenesis of neurodegenerative diseases, thus providing multiple targets for pharmacological intervention [2]. Genetic and pharmacological manipulations designed to induce autophagy have been shown to protect cells in vitro, suggesting the important neuroprotective role of autophagy [3,4]. 

Although many scientists have made great efforts in the treatment of AD, unfortunately, none of existing treatments have been shown to slow or halt the progression of this disease. Currently, some paradoxical pharmacological efficiency has been approved, such as cholinesterase inhibitors and *N*-methyl-d-aspartic acid (NMDA) receptor antagonists, but the long-term application of these drugs can lead to a series of side effects [5]. Thus, novel and effective therapeutic strategies to slow and/or reverse the pathogenesis of patients with AD have gained extensive attention. 

At present, natural products containing polyphenols have gained tremendous interest as the candidates for the prevention and treatment of neurodegenerative disorders. Resveratrol (3,5,4’-trihydroxy-trans-stilbene), a natural polyphenol in grape skin and seeds, can execute a number of physiological and pharmacological functions including anti-inflammatory, anti-oxidative, anti-cancer and cardioprotective functions, and plays an important role in the prevention and treatment of degenerative disorders in brain such as AD [6,7,8]. Considerable evidence suggests that the consumption of synthetic and natural antioxidants as one of the therapeutic strategies has neuroprotective function [9]. Importantly, previous studies have confirmed that resveratrol is involved in several pathophysiological courses of AD [6]. In this review, the data correlated with the molecular mechanisms of resveratrol for regulating autophagy and the regulatory role of resveratrol in microRNAs for modulating autophagy during neurodegenerative diseases have been collected and analyzed. Meanwhile, the current strategies for targeting autophagy and the major issues during the prevention and treatment of neurodegenerative diseases through autophagy as the interventional target have also been discussed. 

## 2. The Regulatory Role of Autophagy in AD

AD is a chronic and progressive dementia. AD patients have difficulty in remembering recent events at the early stage of the disease. During the continuous progress of the disease, AD patients show confusion, irritability, aggression, mood fluctuation and trouble with reading and language [10]. The pathological hallmarks including the deposition of senile plaques and neurofibrillary tangles (NFTs) in AD are due to the loss of hippocampal and cortical neurons. NFTs consist of highly phosphorylated protein Tau, and abnormally phosphorylated Tau is prone to aggregate into paired helical filaments. Therefore, AD can be considered as a continuous decline in cellular and organismal functions due to the accumulation of mis-folded proteins.

### 2.1. Autophagy Change under AD Condition 

It has been reported that signal pathways of autophagy exist in neurons. Neuronal autophagy plays important roles in synaptic plasticity, anti-inflammatory function in glial cells, oligodendrocyte development, and myelination process [11,12]. Functional status of autophagy is vital for healthy organisms, but autophagic activity reveals an obvious decrease in aging organisms [13]. In addition, insufficient or reduced autophagic activity can lead to the formation of harmful protein aggregates and the accumulation of damaged mitochondria, thus correspondingly leading to increased reactive oxygen species (ROS), cell death and neurodegeneration. For example, the overexpression of α-synuclein can cause Parkinson’s disease. On the other end of the process, dementia can be caused by mutations of presenilin-1, endosomal sorting complexes required for transport (ESCRT) machinery or valosin-containing protein (VCP)/p97. The motor neuron disease caused by mutations in the dynein apparatus is associated with decreased delivery of autophagosomes to lysosomes and/or decreased degradation of autophagic substrates in lysosomes [14]. Moreover, previous studies have demonstrated that an aging-related decline of chaperone-mediated autophagy (CMA) receptor in lysosomal membrane can lead to the lower degradation rate of potentially harmful proteins [15]. Meanwhile, impaired autophagy contributing to neurodegeneration is also supported by the disease-like phenotypes, as confirmed in mice with neuron-specific knockouts of autophagy-related genes [16]. Previous studies have shown that abnormal accumulation of autophagic vacuoles including autophagosomes or autolysosmes has been observed in affected neurons of brain in several neurodegenerative diseases. The accumulation of pathological autophagic vacuoles has also been observed in presenilin-1/amyloid precursor protein (PS1/APP) mouse AD model [17]. This phenomenon, as explained by most studies, is due to impaired autophagic flux and degradation, and the resultant decreased proteolysis of Aβ [18]. However, autophagosomes may hardly be detected in healthy neurons under normal nutrient conditions, thereby suggesting that up-regulating neuronal autophagy may provide a protective effect [19]. Additionally, promoting autophagy in *Drosophila* brain can rescue it from neurodegeneration, which suggests that up-regulating autophagy in these circumstances should be beneficial [20]. 

Proteins causing neurodegenerative diseases easily aggregated in intracytoplasm are also autophagy substrates. Soluble monomeric and oligomeric Tau and insoluble Tau aggregates can be cleaved by autophagy process. Recent studies have demonstrated that phosphorylated Tau protein is affected by the failure of autophagy [21]. Beclin1, as one of autophagy markers, is an important controlling mechanism of autophagy and regulates the initiation of autophagosome formation. The autophagy-related Beclin1 level is significantly reduced in brain tissue of AD patients. In addition, the lowered level of Beclin1 can lead to Aβ accumulation and neurodegeneration in a mouse model with AD [22]. Furthermore, the decline of Beclin1 is also obvious in entorhinal cortex and hippocampus, and the further decline can accelerate the progression of AD. Consistent with the early stage, the declined Beclin1 is also detected at the late stage of AD. More recently, the significant decline of Beclin1 has also been confirmed in neurons of AD patients [23]. Additional evidence suggests that Beclin1 reduction impairs phagocytosis in microglia, affects APP processing in neurons, and increases Aβ deposition and neurodegeneration in APP transgenic mouse model with AD [24].

### 2.2. Signal Pathways for the Regulation of Autophagy in AD

Autophagy is tightly regulated by several signal pathways, such as mammalian target of rapamycin (mTOR) signal pathway, as a negative regulator of autophagy. Recent studies indicate that mTOR inhibition can induce autophagy in neurons. For example, rapamycin, as a selective inhibitor of TORC1, attenuates Aβ accumulation and inhibits Tau phosphorylation in AD mouse model [25]. Moreover, metformin treatment inhibits Tau hyper-phosphorylation through inhibiting mTORC1 and is currently tested in clinical trials of AD [26]. Accumulating evidence suggests that the complex consisting of uncoordinated (Unc)-51-like kinase 1 (ULK1) and vacuolar protein sorting (Vps34) is the key regulator for the initiation and progression of autophagy. Moreover, autophagy activation may be achieved by mTOR-independent mechanisms such as activating ULK1 and AMPK, increasing PI3P level, or changing intracellular calcium signals [27].

## 3. Roles of MicroRNAs in AD

MicroRNAs (miRNAs) are small non-coding RNAs with 18–25 nucleotides in length, and can negatively regulate mRNA stability and protein expression by targeting specific mRNAs. Over 70% of reported miRNAs are abundant in brain and play an important role in neurodevelopment and synaptic plasticity [28]. To elaborate which miRNA is important in the production of pro-inflammatory cytokines in AD, mRNA targets and specific roles in neuroinflammation need to be elucidated. There are a large number of brain-specific miRNAs including: miR-134, miR-135, miR-let-7g, miR-101, miR-181a-b, miR-191, miR-124, miR-let-7c, miR-let-7a, miR-29a, and miR-107 [29,30]. Abnormal patterns of miRNA expression have been linked with aging-related diseases including AD [31]. Since neuroinflammation is involved in the process of AD, there are some overlaps between miRNAs in neuroinflammation and AD progression. Therefore, cytokines-associated miRNAs appear to have central roles in both inflammation and AD.

### 3.1. Aβ and MiRNAs 

Some miRNAs have been implicated in governing senescence in a variety of human cell lines, and the precise functions of these miRNAs in regulating cellular senescence have prompted us to explore the underlying mechanisms of aging. Aβ production and Aβ deposits in brain of patients with AD are accompanied by the alteration of many miRNAs from distinct miRNA classes. Previous studies have indicated that miR-101 and miR-106 can target APP, thereby resulting in an elevated generation of Aβ [32,33,34,35]. Some investigations have reported the lower levels of many miRNAs in AD patients and animal models. For example, miR-124 as a neuron-specific miRNA is down-regulated in AD [36]. The down-regulated miR-124 leads to the altered splicing of APP and promotes the transformation of APP into Aβ. Moreover, the decrease of miR-17, miR-101 and miR-16A is accompanied by an increase in APP [33,37]. In addition, the reduced miR-106b expression has been found in sporadic patients with AD and miR-106b may influence APP expression and transforming growth factor beta (TGF-β) signaling, thereby contributing to the pathogenesis of AD [33,38]. Moreover, APP expression can be regulated by miR-17-5p, miR-153 and miR-101 in neuronal cell lines, suggesting that the changes of miR-17-5p, miR-153 and miR-101 may be the important risk factors of cognitive decline in the middle-aged rats. 

### 3.2. Beta-Secretase 1 (BACE1), Tau and AD

Similar to Aβ regulated by miRNAs, altered levels of miRNAs can modulate BACE1 activity. MiR-29c is enriched in brain, however, is down-regulated in sporadic AD brain, and its down-regulation is negatively correlated with BACE1 expression [39]. Interestingly, the overexpression of miR-29c in SH-SY5Y and HEK-293T cell lines and miR-29c transgenic mice reveals the reduced BACE1 expression and the down-regulated APPβ accumulation in vitro [40]. Meanwhile, the up-regulation of miR-29c can promote learning and memory behavior in SAMP8 mice, suggesting that miR-29c may be a possible therapeutic target against AD. During the aging process, miR-186 level is decreased in brain cortices of mice, which is accompanied by an increase in BACE1 in neuronal cells. Therefore, miR-186 is a potent negative regulator of BACE1 in neuronal cells and it may be one of the molecular links between brain aging and the increased risk for AD during aging process [41]. Reduced levels of miR-9, miR-29a, miR-29b and miR-107 may result in the elevated BACE1 expression and an over-production of Aβ known to characterize brain from human and mice with AD [33,42]. In addition, miR-188-3p is significantly down-regulated in brain of human and its transgenic (TG) mice with AD. However, the overexpression of miR-188-3p in hippocampal tissues of TG mice results in the reduced BACE1, Aβ and neuroinflammation [43]. In patients and mice with AD, miR-328, miR-298, miR-339-5p, miR-384 and miR-107 also can regulate the expression of BACE1 [44,45,46,47].

The alteration of miRNAs may have effect on Tau hyper-phosphorylation. For example, the loss of miR-15a facilitates the hyper-phosphorylation of Tau by disinhibiting ERK1 [48,49]. However, up to date, the studies on miRNA functions in human aging are still rare. Further studies are needed to better understand the involvement of aging-related miRNAs in AD pathogenesis. Thus, the modifications of dys-regulated miRNAs in brain are proposed as the putative biomarkers of these aging-related diseases. 

## 4. Interplay between MiRNAs, Autophagy and AD

In recent years, accumulating miRNAs have also been reported to be involved in autophagy modulation by regulating the expression of autophagy-related genes. Indeed, miRNAs are shown to change the levels of several key proteins in the process of autophagy including the upstream signal pathways of autophagy, and the initiation, elongation and fusion of autophagy as well as the autolysosomal degradation. 

Among autophagy-related miRNAs, many of them are involved in the early stage of AD. Others are involved in the late stages of diseases. These miRNAs are shown to result in the impact on Tau phosphorylation, protein aggregation and neuroinflammation. Furthermore, some autophagy-related miRNAs are identified as the biomarkers or therapeutic targets of AD. Especially, the effects of miRNAs on autophagy-related genes and/or proteins are critical for AD-related outcomes in many studies. Therefore, targeting these miRNAs or miRNA-related components in the autophagic degradation system may be decisive in the control of AD progression.

## 5. The Neuroprotective Effect of Resveratrol on AD through Regulating Autophagy and MiRNAs 

As abnormal protein aggregation cause most neurodegenerative diseases, it is urgent to develop effective and innovative therapeutic strategies with the function of enhancing the degradation of these toxic aggregates. Considering its function of primarily degrading abnormal proteins and/or organelles, autophagy is considered as an emerging therapeutic target so that exploring autophagy inducers should have the potential to become an ideal strategy for the prevention and treatment of AD. 

Considering that autophagy induction may prevent neurodegenerative diseases, dietary interventions seem interesting. Epidemiological studies have indicated that appropriate consumption of polyphenol-rich foods may be associated with a lower risk of chronic diseases. Resveratrol, as a natural polyphenol rich in red wine, has multiple biological functions including anti-inflammatory, antioxidant and neuroprotective functions in vitro. The appropriate consumption of red wine can attenuate clinical dementia produced by AD in human [50]. Currently, there are several lines of evidence underlining the neuroprotective effects of resveratrol. Resveratrol treatment prevents neurodegeneration and cognitive decline in mice displaying AD features [51,52]. Consistent with a previous study, resveratrol has shown a memory improvement by increasing antioxidant activity in AD rats [53]. Furthermore, resveratrol can attenuate Aβ-induced cytotoxicity, cell apoptosis, thereby increasing cellular viability in Aβ-induced PC12 cells [54]. With the research advancement, mounting evidence has shown that resveratrol treatment can markedly decrease the levels of secreted and intracellular Aβ peptides by facilitating Aβ proteolytic clearance in different cell lines and primary neurons [55], and reduce the amyloid deposition in brain tissues of APP-transgenic mice [51,56]. In addition, resveratrol is known to have beneficial metabolic effects and is considered a mimetic of dietary/caloric restriction. Previous reports have demonstrated that caloric restriction can attenuate β-amyloid neuropathology and improve glucose metabolism in a mouse model of AD [57]. 

### 5.1. Resveratrol-Induced Activation of Autophagy through Different Signal Pathways

**Figure 1:**
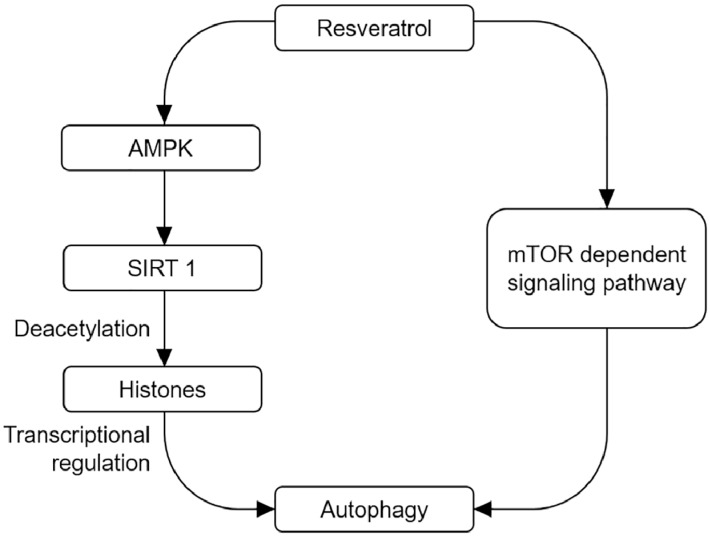
Autophagy-mediated neuroprotective effects by resveratrol. On the one hand, resveratrol can activate SIRT1 and deacetylate histone acetylases through AMPK/SIRT1 signal pathway. On the other hand, resveratrol can directly activate autophagy and exert neuroprotective effect by regulating mTOR signal pathway.

The up-regulation of autophagy appears to decrease the susceptibility to pro-apoptotic insults, which may have further benefits [58]. Thus, the up-regulation of autophagy mediated by a wide range of compounds can enhance the clearance of aggregation-prone proteins, and such compounds can attenuate the toxicity in different disease models [58,59,60,61,62]. Resveratrol has been suggested to modulate cellular processes by activating key metabolic sensors/effectors, including AMP-activated protein kinase (AMPK), sirtuin 1 (SIRT1), and peroxisome proliferator-activated receptor γ co-activator-1α (PGC-1α) [56,63]. Resveratrol can execute the impact on mitochondrial functions, and also can act as an activator of SIRT1, increase NAD^+^/NADH ratio, and enhance the clearance of mutant proteins associated with neurodegenerative diseases via mTOR-dependent or independent manner to promote neuronal survival [64]. Resveratrol has also been reported as the anti-aging agent to enhance lifespan through pro-autophagic mechanisms [65,66,67,68]. In addition, AMPK is a Ser/Thr protein kinase predominantly expressed in neural tissue and is activated by different upstream kinases such as Ca^2+^/CaM-dependent protein kinase kinase β (CaMKKβ). Resveratrol is a potent activator of AMPK in cultured cells or mice [56,69]. Moreover, resveratrol and its analogs, resveratrol A314 and resveratrol A405, show protective effect against AD by activating AMPK. Mechanically, resveratrol activates AMPK by increasing intracellular Ca^2+^ levels and promoting AMPK phosphorylation at Thr172 site, thus resulting in mTOR inhibition and increasing autophagic and lysosomal clearance of Aβ. However, recent studies have reported that there are direct interaction between resveratrol and SIRT1 in vitro [70,71]. SIRT1, AMPK and mTOR signal pathways are involved in the pathogenesis of AD. Based on the above evidence, resveratrol should have the therapeutic potential of AD through activating autophagy via controlling SIRT1-mediated transcriptional regulation or mTOR-dependent signal pathway (Figure 1). More importantly, resveratrol via oral administration can pass through blood–brain barrier, which will motivate the exploration of resveratrol metabolites/analogues with improved potency and brain penetration properties as anti-neurodegenerative molecules. 

### 5.2. Resveratrol-Mediated Neuroinflammation Change in AD

The pro-inflammatory phenotype of senescent cells, coupled with the up-regulation of inflammatory responses with increasing age, has been found to play an important role in the initiation and progression of aging-related diseases including AD [72,73]. During the aging process of brain, an imbalanced status between pro- and anti-inflammatory cytokine levels including elevated pro-inflammatory cytokines and reduced anti-inflammatory mediators [74]. The presence of Aβ is associated with neuronal inflammation [75]. The neuroinflammation is the hallmark of neurodegenerative diseases. Thus, targeting signal pathways associated with neuroinflammation should be an ideal strategy for the prevention and treatment of neurodegenerative diseases. 

Aging results in a significant increase in glial activation and inflammatory mediators. It is widely accepted that microglia-mediated neuroinflammation plays an important role in the pathogenesis and progression of neurodegenerative diseases [76]. Thus far, the reason for increased inflammation during aging process is not clear. Accumulating evidence strongly suggests that phytochemicals may exert anti-inflammatory activity and antioxidant effect in the context of brain aging. Resveratrol administration can execute neuroprotective functions by scavenging free radicals and suppressing glial activation [77,78]. The NF-κB signaling is an inducer of inflammatory responses and several studies have demonstrated that NF-κB signaling is activated during aging process [79,80]. Resveratrol treatment can suppress Aβ-induced activation of NF-κB in PC12 cells, suggesting a possible therapeutic function of resveratrol in mediating neuroprotection [54]. Recent studies have reported that resveratrol significantly attenuates lipopolysaccharide (LPS)-induced production of inflammatory cytokines such as IL-1β and TNF-α induced by LPS or Aβ in microglia [81,82]. Resveratrol also exerts neuroprotection against hypoxia-induced neurotoxicity by inhibiting AP-1 and degrading IkappaB-alpha (IκB-α) and phosphorylating p65NF-κB. In addition, resveratrol can suppress the activation of c-Jun N-terminal kinase (JNK) and its upstream MAPK signaling cascades [83]. In parallel with previous studies implicating the anti-inflammatory role of resveratrol, resveratrol has also been confirmed to increase the release of anti-inflammatory IL-10 [84,85].

### 5.3. Resveratrol-Mediated MiRNA Change in AD

**Figure 2:**
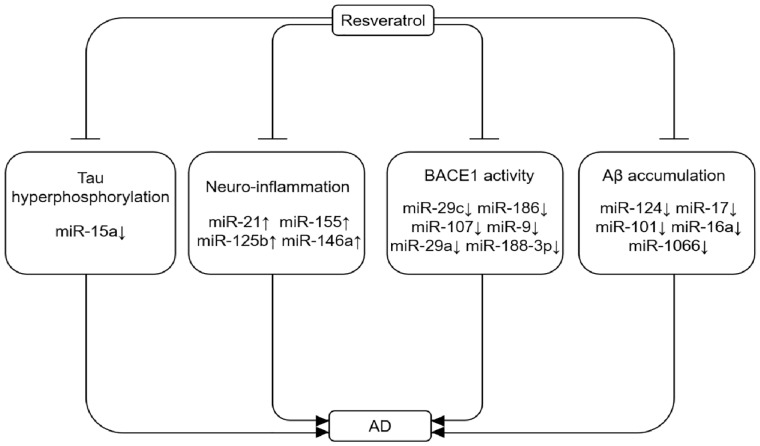
Resveratrol rescues abnormal expression of miRNAs during AD progression. Abnormal expression of miRNAs can lead to Aβ accumulation, Tau hyper-phosphorylation, neuroinflammation, and reduced BAECE1 activity, thereby aggravating AD.

MicroRNAs have been reported to regulate the innate and adaptive immune responses. Specially, miR-21, miR-155, miR-125b and miR-146a are found to be markedly up-regulated in neurodegenerative diseases, which may aggravate neuroinflammation [86,87]. In another study, miR-155 has been confirmed to be a critical pro-inflammatory miRNA for the innate immune response, and the increased expression of miR-155 is observed in neurodegenerative disorders [88]. Moreover, miR-155 is a multifunctional miRNA in AD pathogenesis with a distinct expression profile and link to T-cell functions [89]. Resveratrol has been shown to exert neuroprotective effect through its anti-inflammatory and antioxidant properties by modulating miRNAs (Figure 2). Recent studies indicate that resveratrol can attenuate the up-regulation of pro-inflammatory miR-155 by LPS in a miR-663-dependent manner [90]. In contrast, resveratrol treatment can up-regulate an anti-inflammatory miR-663 in human THP-1 monocytic cells and human blood monocytes. 

Since a large number of miRNAs have been identified in AD, the investigation of miRNAs is a promising in this relatively unexplored area for understanding the regulatory mechanisms of AD. Clearly targeting these miRNAs may provide an efficient, convenient and noninvasive approach for the diagnosis, prevention, or treatment of AD through regulating pro-inflammatory and anti-inflammatory factors.

## 6. Resveratrol and Clinical Trials

According the recent limited clinical trial reports, as shown in Table 1, resveratrol can impact neuroinflammation and Aβ deposition upon the administration of resveratrol for patients with mild or moderate AD [91]. The reduced Aβ40 is determined in both cerebral spinal fluid (CSF) and plasma to a lesser extent in the resveratrol treatment group when compared with the placebo group, suggesting drug penetration to blood–brain barrier to reveal the central effect. In addition, the 52-week phase-II clinical trial of resveratrol in individuals with mild to moderate AD is conducted to exhibit the consistent result with reduced levels of Aβ40 in CSF and plasma upon the administration of resveratrol when compared with the placebo [92]. A recent study has also demonstrated that resveratrol supplementation is associated with favorable immune and cognitive responses in patients with ApoE ε3/ε3 in comparison to those with ApoE ε3/ε4 [93], which suggests a relationship between the improvement of innate immunity and cognitive capacity. More importantly, the most common adverse effects such as nausea, diarrhea and weight loss from resveratrol in participants during clinical trials are similar to placebo, which confirmed that the oral administration of resveratrol at high dose is safe and well tolerated. Although the validation from above recent studies is limited due to its small size, these investigations as the novel and effective intervention strategies also provide the basis and reference for the prevention and treatment of AD. Further studies are highly desired to explore the targets of resveratrol or monitor the change in biomarkers associated with these targets for executing the prevention and treatment of AD during the application process of resveratrol.

## 7. Conclusions and Future Perspectives

With the accelerated race of aging population, AD continues to be a growing health concern worldwide. Although resveratrol has shown significant anti-tumor effects in many studies, its therapeutic effect in AD is also emerging. In general, inducing autophagy is a widely accepted innovative strategy against neurodegenerative diseases. There is a growing body of evidence indicating the properties of resveratrol to counteract AD possibly through modulating AMPK-SIRT1-mediated autophagy signal pathway or miRNA-mediated signal pathways for controlling Tau hyper-phosphorylation, neuroinflammation, BACE1 activity, and Aβ accumulation based on the regulatory roles of miRNAs in AD (Figure 2). However, the precise molecular mechanisms and the direct molecular targets of resveratrol for regulating autophagy remains largely unclear, and future studies should explore the therapeutic potential of resveratrol in vivo models. Concerning miRNAs, a series of questions regarding miRNAs in AD brain need to be further resolved; for example, which specific miRNA is involved in cellular changes associated with neurodegenerative diseases and aging, and whether AD can be prevented or delayed by strategic alterations of miRNA expression.

## Figures and Tables

**Table 1:** Clinical trials of resveratrol (RSV) in AD.

Participants	Dosage	Duration of Treatment	Key Outcomes	References
119	500–1000 mg once daily	52 weeks	RSV-induced improvement of brain volume; RSV is safe and well tolerated.	[92]
119	500–1000 mg once daily	52 weeks	RSV-induced reduced Aβ in plasma and CSF as well as declined MMP.	[91]
18	150 mg	48 weeks	RSV-induced improvement of cognition and innate immune functions.	[93]
